# Recognition of rotated objects and cognitive offloading in dogs

**DOI:** 10.1016/j.isci.2022.103820

**Published:** 2022-01-26

**Authors:** Lucrezia Lonardo, Elisabetta Versace, Ludwig Huber

**Affiliations:** 1 Comparative Cognition, Messerli Research Institute, University of Veterinary Medicine Vienna, Medical University of Vienna and University of Vienna, 1210, Vienna, Austria; 2 School of Biological and Behavioural Sciences, Department of Biological and Experimental Psychology, Queen Mary University of London, E1 4NS, London, UK

**Keywords:** Canine behavior, Behavioral neuroscience, Cognitive neuroscience

## Abstract

Recognition of rotated images can challenge visual systems. Humans often diminish the load of cognitive tasks employing bodily actions (cognitive offloading). To investigate these phenomena from a comparative perspective, we trained eight dogs (*Canis familiaris*) to discriminate between bidimensional shapes. We then tested the dogs with rotated versions of the same shapes, while measuring their accuracy and head tilts. Although generalization to rotated stimuli challenged dogs (overall accuracy: 55%), three dogs performed differently from chance level with rotated stimuli. The amplitude of stimulus rotation did not influence dogs’ performance. Interestingly, dogs tilted their head following the direction and amplitude of rotated stimuli. These small head movements did not influence their performance. Hence, we show that dogs might be capable of recognizing rotated 2D objects, but they do not use a cognitive offloading strategy in this task. This work paves the way to further investigation of cognitive offloading in non-human species.

## Highlights

• Dogs have the potential to recognize 2D objects even when these are rotated
• After training, females were more accurate in discriminating upright images
• Dogs tilted their head in the same direction as the images were rotated
• In our setting, dogs did not offload a cognitive process onto their bodies

## Graphical abstract

**Figure:**
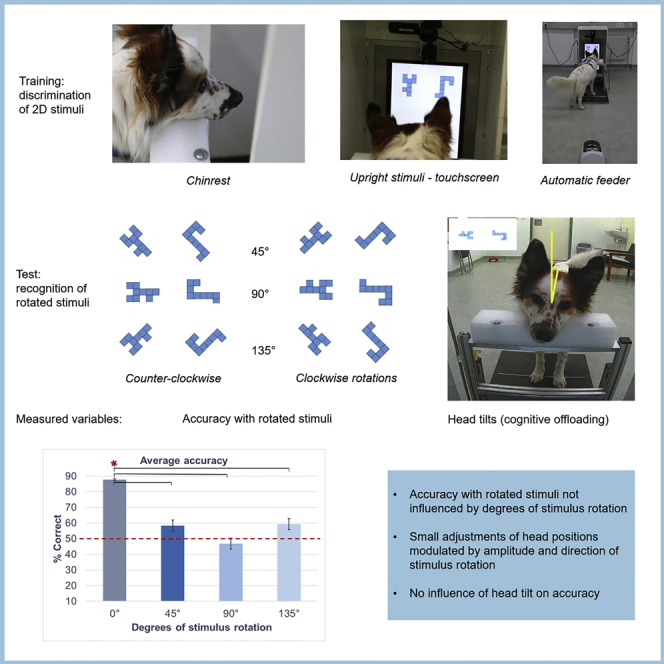

## Introduction

When engaging in mental tasks, we spontaneously try to overcome the limitations of our cognitive systems by transferring part of the intellectual demands onto our bodies or onto the external world. This strategy is known as cognitive offloading (for a review of the human literature see Risko and Gilbert 2016). Little is known on cognitive offloading in non-human animals. Are physical actions that reduce mental load a uniquely human strategy or a behavior shared across species? To answer this question, we assessed whether pet dogs (*Canis familiaris*) spontaneously try to decrease the cognitive load of a mental rotation task by tilting their heads in the same direction as the stimuli, like humans do.

Mental rotation has been hypothesized as a cognitive process used by humans to recognize an object seen from an orientation different than a reference stimulus, by mentally rotating and aligning the internal (mental) representation of the object with the reference (e.g., Shepard and Metzler, 1971; Cooper and Shepard, 1973). However, the mental rotation account has been challenged and may be outdated. In particular, it seems that this account is best suited to explain results from tasks requiring to judge whether two objects are identical or mirror images of each other (Gauthier et al., 2002). Several alternative models have been proposed to explain how the human visual system recognizes whether two objects seen from different points of view are the same or not. For example, some researchers proposed that object recognition might be based on more than just one processing mechanism (the “multiple routes” hypothesis, Vanrie et al. (2001)) or that it might be based on interpolation between the limited views of an object stored in memory (Edelman and Poggio, 1991; Riesenhuber and Poggio, 2000). The strategies used by non-human species to recognize rotated objects are debated too, as discussed below.

The cognitive load of recognizing the rotated stimuli in the generalization task can be reduced by physical actions that reduce the degree of mismatch between the test and the training stimulus, such as tilting the head (Risko and Gilbert, 2016). In humans, cognitive offloading is common in different domains: people use reminders for future events, rotate maps to match one’s own point of view, count with the help of fingers, write things down, and tilt their head during the perception of ambiguous images (Carlson et al., 2007; Chu and Kita, 2011; Gilbert, 2015; Goldin-Meadow et al., 2001; Risko et al., 2014). Indeed, cognitive offloading strategies improve performance also for recognition of rotated objects, for instance by increasing the speed of reading rotated text (Risko et al., 2014).

Two lines of evidence support the idea of an analog representation and mental rotation model in humans. First, a larger number of mistakes when the object and the reference have a greater angular disparity (Bauer and Jolicoeur, 1996; Delius and Hollard, 1995; Foster, 1978; Hall and Friedman, 1994; Hollard and Delius, 1982; Parsons, 1987; Wohlschläger, 2001; Wohlschläger and Wohlschläger, 1998). Second, a near-linear increase in latency with rotation difference. In humans, the increment in reaction times has been interpreted as the effect of an analog mode of visual information processing in which an object’s mental representation is transformed in a serial, time-consuming process (Shepard and Metzler, 1971; Stich et al., 2003). In a classical study, Shepard and Metzler (1971) investigated how humans decide whether two differently rotated objects are the same or not. Eight participants were presented with pairs of differently rotated drawings of solids, a reference, and a target. They judged whether the two objects were the same but rotated or whether they were different objects (mirror images that could not be rotated into congruence). Rotations consisted of 20° steps, from 0° to 180°. On average, only 3.2% of the responses were incorrect (ranging from 0.6% to 5.7% for individual subjects). The time used to make the judgment was a linear function of the angular disparity between the two figures. Based on this result, the authors described mental rotations as an analog transformation process of image-like representations of visual information.

Neuroimaging data have supported both the analog representation view and the hypothesis that mental rotation depends on motor simulation, i.e., the planning of motor processes (Zacks, 2008). Mental rotation could be considered an imagined (covert) action or at least partly produced in conjunction with the motor system (Lamm et al., 2007; Wexler et al., 1998; Wohlschläger, 2001).

With analog mental representations, the cognitive load imposed by the effort of mental rotation can be reduced either by rotating the objects in the external world or by tilting the head of the observer. The conditions under which humans engage in cognitive offloading while processing rotated stimuli have been investigated by Risko et al. (2014). In these experiments, participants were asked to read rotated letters and text. This task demands “normalization” of the viewpoint, i.e., alignment of the rotated stimuli to their canonical orientation. When presented with sets of 1, 5, or 15 rotated letters, participants spontaneously exhibited head tilts on approximately 16% of the trials but this tilting did not improve nor hinder their reading accuracy. Head tilts were exhibited more frequently with increasing set size (and hence increasing mental effort required by the task). Indeed, participants tilted their head on average on 3% of the trials presenting only 1 letter, on 18% of the trials presenting 5 letters, and on 37% of the trials presenting 15 letters. Risko et al. therefore showed that human head tilts can be systematically investigated as an instance of cognitive offloading in a controlled environment.

While cognitive offloading has been extensively investigated in humans, much less is known on non-human species. Pigeons (*Columba livia*) can discriminate mirror-image shapes equally fast and well regardless of orientation disparities, a skill known as orientation invariance, presumably based on a parallel mode of information processing (Hollard and Delius, 1982). Even highly intelligent humans could not match the birds' performance. Several types of stimuli did not lead to a rotation effect in pigeons: novel mirror-image stimuli, rotation of sample shapes, a delayed display of comparison shapes, and a mixed use of original and reflected sample shapes (Delius and Hollard, 1995). Interestingly, with misaligned arbitrary shapes, humans failed to show a mental rotation effect, similarly to pigeons. This finding led Delius and Hollard (1995) to conclude that the complete absence of a rotation effect in pigeons is due to an advantage in discriminating mirror-image shapes compared with arbitrary shapes. It is possible that humans perceive the orientation differences of arbitrary shapes but are not obstructed by them in the same way as when discriminating mirror-image shapes.

Evidence coming from the arboreal living lion-tailed macaque seems to hint at a hybrid status of monkeys’ information processing mode. Indeed, these macaques recognized non-rotated stimuli faster than rotated ones, but showing no clear relationship between reaction times and angle of rotation (Burmann et al., 2005). The testing of a more terrestrial living Rhesus monkey has also yielded inconsistent results, with some evidence for both processes, mental rotation and rotational invariance (Köhler et al., 2005), supporting the view of two separately evolved information processing systems that may be coexisting to a certain extent in species with correspondingly overlapping ecological demands.

Delius and Hollard (1995) have speculated on why humans do not benefit from the potential rotational invariance capability of the primate visual system by suggesting bio-evolutionary adaptations due to special demands of the lifestyle. While pigeons operate visually on the horizontal ground plane both in flight and walking, humans, who have abandoned the arboreal lifestyle of our primate ancestors, mainly operate visually on the vertical plane. Owing to an upright gait, humans mostly see the environment in the vertical plane and therefore are used to a rather restricted number of environmental perspectives. If orientation invariance is neurally elaborate and costly, humans might have secondarily lost it. The fact that pigeons—a species lacking hands or similar effectors allowing continuous object rotation—are not affected in their discrimination performance by the rotation of stimuli has also been interpreted as consistent with the view that the motor system might play a role in the mental rotation process (Wohlschläger and Wohlschläger, 1998).

In non-human animals, no study has investigated the link between mental rotation and cognitive offloading. The only study with at least an implicit answer to this question was by Hollard and Delius (1982) who reported that, while recognizing rotated images, pigeons rarely inclined their heads by more than 30° and that the head position did not relate in any obvious way with the orientation of the forms. However, pigeons might benefit from a kind of parallel processing, with which they achieve rotational invariance; thus, they might have no need for facilitating the internal transformation to bring the mental representation into alignment with the object.

Here we focus on pet dogs, to clarify whether a species that has lived in the same household as humans for more than 14 thousand years (Janssens et al., 2018) has evolved similar capacities for recognizing rotated objects and cognitive offloading. Based on the shared evolutionary history of ecological constraints on perception between dogs and humans, we expected dogs to recognize rotated stimuli similarly to humans. Therefore, we predicted that their accuracy should have decreased with increasing angular disparity between probe and reference. In our setting, it was not possible to measure dogs’ reaction times because our main focus was observing their possible head tilts prior to choice. Hence, we let dogs wait a fixed time interval before allowing them to give a response.

To date, apart from humans, no purely terrestrial mammal has been tested for their ability to generalize to rotated stimuli. An interesting mammalian model is the domestic dog, as these animals do not only see a very similar environment as their human caregivers but they also perceive many objects in the vertical plane due to gravity.

Domestic dogs have been tested in many visual tasks, ranging from simple (e.g., Milgram et al., 1994; for a comprehensive review see Bensky et al., 2013) to more complex discriminations. At a larger scale, they have proven the ability to discriminate between visual classes, such as dog and landscape images, according to a perceptual response rule (Range et al., 2008). In sum, dogs can clearly learn to discriminate between various arbitrary stimuli based on differential reward contingencies, as reviewed in Byosiere et al. (2018). In the present study, we trained our dogs on a visual discrimination task, in which they had to distinguish between two abstract geometrical shapes having the same area and color.

Here, we modified for dogs an object recognition task to test the ability of pet dogs to first acquire a discrimination of two geometrical shapes and then transfer this ability to rotated versions of the same shapes. By looking at the number of errors in relation to different degrees of stimulus rotation, we aimed at understanding whether dogs exhibit a human-typical performance (lower performance at larger rotation distance) or a pigeon-like rotation invariance performance. If dogs are able to discriminate between the two shapes when these are presented at rotation angles different from the training one, evidence that the task is solved using a human-like strategy would be that (1) they are more accurate for angles of stimuli rotation closer to the training orientation and (2) they exhibit wider head tilts for greater stimuli rotation angles, in an attempt to relieve the increasingly demanding cognitive process.

The second aim of the study was to examine the use of cognitive offloading with rotated stimuli. Only recently, Sommese et al. (2021) found a relationship between head tilting and the processing of auditory stimuli, but only if those had been relevant and meaningful. From their data, the authors concluded that head tilts are a sign of increased attention. Dogs might tilt the head also as a kind of external way of alignment instead of rotating an internal (mental) representation of the presented stimulus before making the judgment. If the mechanism that transforms an input shape into the orientation of the presented shape is cognitively demanding, dogs might use the cognitive offloading strategy to simplify the task.

A third aim of the experiment was to explore sex and individual differences. A study investigating how dogs respond to a violation of size constancy (Müller et al., 2011) found surprisingly large sex differences. Female dogs looked significantly longer when the size of a rolling ball seemed to “magically” change after rolling temporarily behind a barrier while males did not. The authors suggested the existence of cognitive differences between sexes in their task is a by-product of other sex differences in spatial cognition. Building on this finding, we were interested in testing whether sex differences would have emerged in this mental rotation task as well and, if so, if they would have emerged in the same direction, with females outperforming males. Moreover, not only individual differences in performance but also in the adoption of different strategies in solving the mental rotation task have been found with Rhesus monkeys (Köhler et al., 2005). Similarly, we expected to find large inter-individual variation in dogs too and hence have analyzed each subject’s performance separately.

## Results

### Learning curves

The tested dogs needed between 15 and 127 sessions to learn the visual discrimination task (see Table S12 for individual results). The huge inter-individual variability in learning speed is portrayed in Figure S1.

### Accuracy during training

The sample for this model comprised 23 dogs, trained over 1159 sessions for a total of 22,919 trials. The number of correct choices was 13,621, while the number of incorrect choices was 9298 (see Table S1 for each of the tested dogs’ accuracy during training). Each dog was trained for at least 30 sessions. The vast majority of sessions consisted of 20 trials. However, a minority of sessions were terminated before the dog completed all 20 trials. This happened mainly due to system malfunctioning or if the dog stopped spontaneously approaching the touchscreen.

Overall, there was no effect of sex nor of its interaction with session number on the proportion of correct choices (likelihood ratio test comparing full and null model: χ^2^ (2) = 1.526, p = 0.466). None of the interactions was significant, as shown in Table S2.

There was no main effect of sex on accuracy and the interaction between sex and session number was not significant either. There was no main effect of age on accuracy and the interactions between age and age squared and session number were not significant either (Table S3). This suggests that, during training, there was no difference in the performance of dogs (both females and males) of all ages (from 5 months to 14 years), who reached similar levels of accuracy. The only significant effect was that of session number (Table S4). As the number of training sessions increased, dogs became significantly more accurate in discriminating between the two upright shapes.

### Accuracy during test

Dogs were tested on a total of 3264 upright trials (rotation: “none”) and on 576 rotated trials (half of which with clockwise rotations). See Table S5 for each of the 8 tested dogs’ accuracy with upright and rotated stimuli and Table S6 for their performance with each angle of rotation.

Because the full-null model comparison was significant (χ^2^ = 6.993, Df = 2, p value = 0.030), we further proceeded in testing the significance of the individual predictors on accuracy.

First, direction of rotation had a significant influence on accuracy (χ^2^ (2) = 17.127, p value < 0.001). This effect was due to a significantly worse performance with rotated stimuli relative to upright stimuli (Table S6). Indeed, all dogs were less accurate with rotated stimuli than with upright stimuli. The proportion of correct responses averaged among the 8 subjects was approximately 88% for upright stimuli and 55% for rotated stimuli.

**Figure 1:**
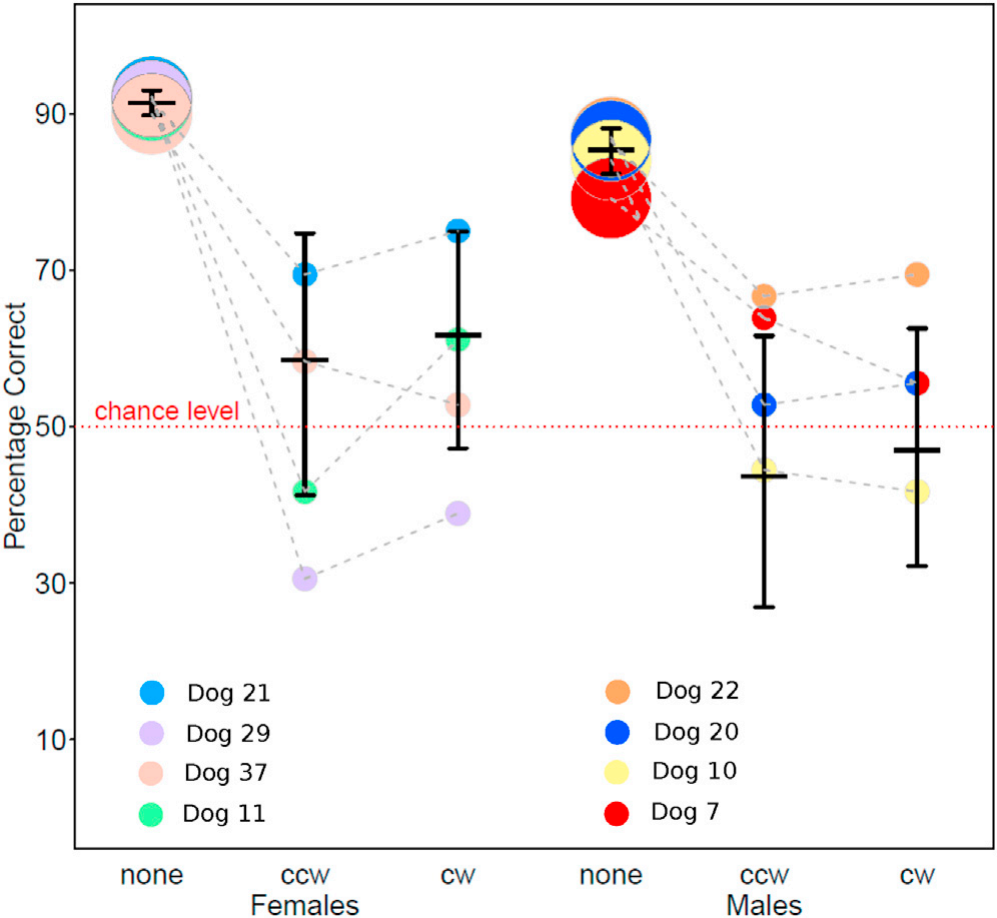
Accuracy during test Percentage of correct responses for each sex and direction of stimulus rotation (“none” indicates performance with upright stimuli, “ccw” with counter-clockwise rotated stimuli and “cw” with clockwise rotated ones). Females are represented on the left, males on the right. The horizontal black lines indicate the fitted values and the error bars refer to their CIs. Each colored bubble indicates the mean percentage of correct responses of an individual dog with each rotation (each dog is shown with the same color across conditions). The area of the bubbles is proportional to the number of observations per dog and condition. The gray dashed lines connect the observations of the same individuals across conditions. The red dotted line shows the chance level. See also Table S6.

Second, there was no effect of the angle of stimulus rotation on accuracy (Table S6, predictor “Rotation”). Hence, within the rotated stimuli, the wideness and direction of rotation (±45°, ±90°, ±135°) did not have an influence on performance (Table S6 and Figure 1). This means that performance was similarly inaccurate for smaller and wider stimulus rotations. We additionally compared the accuracy with clockwise and counter-clockwise rotated stimuli using a Wald test. This confirmed that performance was similarly inaccurate for clockwise and counter-clockwise rotations (pairwise comparison ccw-cw rotations: z = 0.342, p value = 0.732).

Third, we found an effect of sex on performance, with females being significantly more accurate than males. As in the training phase, age did not have an influence on performance. Finally, there was an effect of session number on accuracy: with increasing session number, accuracy increased as well.

A post hoc analysis of solely the trials that presented rotated stimuli revealed no significant effect of session number on performance (estimate ±SE = 0.085 ± 0.087, χ^2^ (1) = 0.948, p value = 0.330). Therefore, over the course of the 24 test sessions, dogs improved their performance in rewarded trials (upright stimuli) but not in unrewarded trials (rotated stimuli). Interestingly, analyzing the performance with rotated stimuli only, the sex effect disappeared (estimate ±SE = χ^2^ (1) = 0.522, p value = 0.470) too, meaning that females were more accurate than males only with upright reinforced stimuli but not with the novel, rotated, and unrewarded stimuli.

The estimate of stimulus rotation was approximately 0.02, while the estimated standard deviations among reinforced stimulus varied (depending on the random slope within reinforced stimulus) to a maximum of 0.61. This indicates that the estimated variation among different reinforced stimuli was large compared to that among different angles of stimuli rotation.

### Head tilts and their influence on performance

The average head tilt after the stimulus onset was 4.66° wide (SD = ± 3.94) and we observed heads being inclined by more than 10°, after the stimulus onset, in only 43 of 508 trials.

At a group level, we found a significant effect of the degrees of stimulus rotation on head rotations measured after the stimulus onset (χ^2^ (1) = 5.48, p = 0.019). This means that, as expected, head rotation was influenced by stimulus rotation in a linear fashion, with dogs rotating their heads in the same direction as the stimulus rotation (clockwise or counter-clockwise) and with wider head tilts in response to wider stimulus rotations. We did not find any effect of sex on the wideness of head tilts (χ^2^ (1) = 0.29, p = 0.589), as shown in Figure 3 (see also Table S7). We found that only one dog’s (subject 29) wideness of head tilts was linearly influenced by the angle of stimulus rotation (see Table S10 and Figure S2).

**Figure 2:**
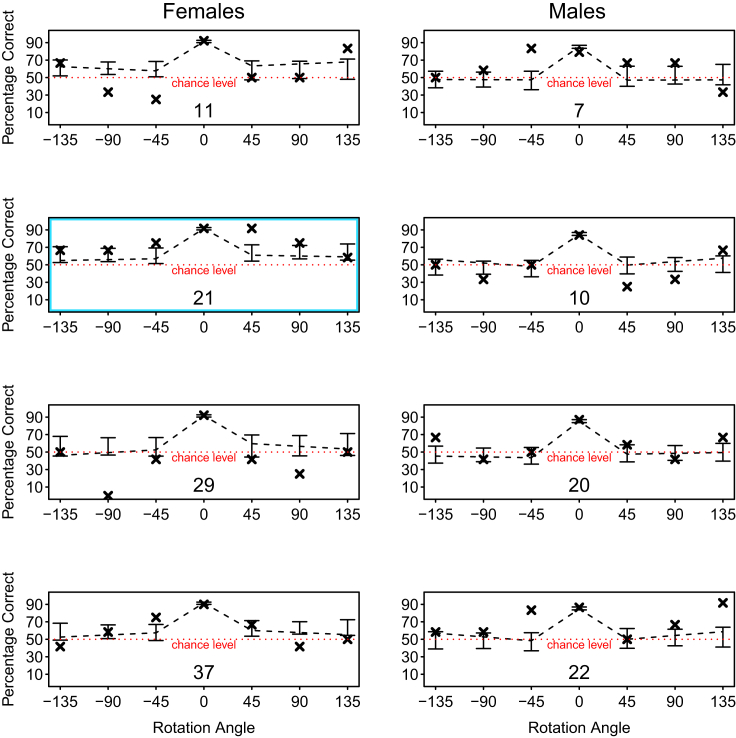
Individual’s performance as a function of the angle of stimulus rotation Females are shown in the left column (from top to bottom: dog 11, 21, 29, and 37), males in the right column (top to bottom: dog 7, 10, 20, and 22). The crosses indicate the mean observed performance; the dashed line indicates the fitted values. Error bars represent the CIs of the fitted values. Negative numbers on the x axis refer to counter-clockwise rotations, while positive numbers refer to clockwise rotations. Dog 21 (second plot of the left column, outlined in blue) achieved significantly above chance level performance with all rotations. The chance level is indicated by the red dotted line. See also Table S5.

**Figure 3:**
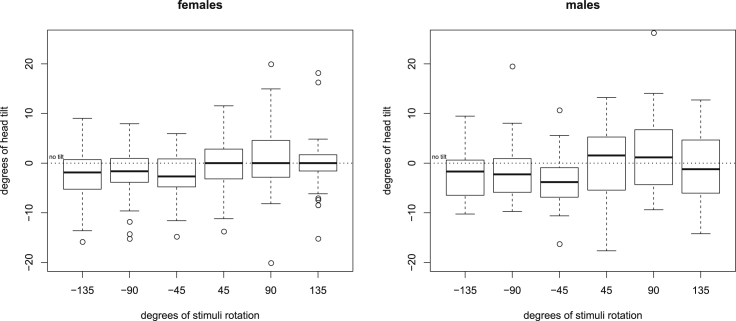
Degrees of head tilt as a function of stimulus rotation On both the x- and the y axis, negative and positive numbers refer to counter-clockwise and clockwise rotations. The y axis shows the degrees of head rotations measured after stimulus onset. Data are represented as median ± IQR. The upper whiskers extend from the 3rd quartile to the largest value or no further than 1.5 ∗ IQR from the 3rd quartile. The lower whiskers extend from the 1st quartile to the smallest value or not below 1.5 ∗ IQR from the 1st quartile. Outliers are represented as individual points outside the whiskers of the boxplots. See also Figure S2 and Tables S7 and S8.

Overall, there was no effect of the test predictors on the difference between dogs’ head rotations after and before the stimulus onset (likelihood ratio test comparing full and null model: χ^2^(2) = 1.51, p = 0.469). Likewise, in contrast to the previous model, we did not find an effect of the angle of stimulus rotation when we used the difference between head rotation after and before the stimulus onset as dependent variable (χ^2^(1) = 1.512, p = 0.219). Similar to the previous model, sex did not affect (χ^2^(1) = 0.001, p = 0.973; see Table S8).

Overall, there was no effect of the test predictors on the proportion of correct choices (likelihood ratio test comparing full and null model: χ^2^(2) = 0.64, p = 0.727). Likewise, the absolute difference between the degrees of head tilt and the degrees of rotation of the stimuli and sex had no influence on performance (Table S9). These results suggest that the wideness of head tilts did not have any influence on accuracy.

## Discussion

Little is known on the link between the recognition of rotated objects and cognitive offloading in non-human animals. We investigated how dogs process rotated stimuli by analyzing their generalization performance with rotated versions of the training stimuli and their head tilts as a means to ease the cognitive load of the seemingly difficult task.

Overall, dogs’ generalization performance with rotated stimuli decreased compared to their performance with upright stimuli. The overall performance with rotated stimuli was significantly different from chance level for three (38%) of the eight tested dogs: significantly above chance level for two dogs and significantly below chance level for one dog. These results show that dogs are capable of solving a task similar to those that humans solve with mental rotation.

We investigated whether dogs recur to external normalization (head tilting) while observing rotated stimuli from a fixed position on a chinrest. We found that, while using a chinrest, dogs tilted their heads very rarely. They made small adjustments in their head positions after stimuli were presented. However, the angle of stimulus rotation had a linear influence on dogs’ head tilts. At the individual level, head rotations of only one dog (dog 29, the one performing significantly below chance level in the recognition of rotated objects task) were linearly associated with the angle of stimulus rotation. Overall, this evidence suggests that dogs might use a cognitive offloading strategy. However, the effect of stimulus rotation on head tilts was not significant when we considered as dependent variable the difference in head tilt between after and before the stimulus onset. This might be due to the fact that dogs straightened their head out for smaller rotations of the stimuli but left their head tilted for wider stimulus rotations. In any case, head tilting did not impact accuracy with rotated stimuli.

It has been proposed that differences in the visual information processing systems across species might have emerged in response to the ecological and evolutionary demands of adapting to different ecological niches (Delius and Hollard, 1995; Köhler et al., 2005). Given that dogs have massively shared their habitat with humans, we expected similarities in the strategies used by the two species in this experiment. In different studies involving mental rotation and recognition of rotated objects, human participants have shown a tendency to commit more mistakes as angular disparity increased (Bauer and Jolicoeur, 1996; Delius and Hollard, 1995; Foster, 1978; Hall and Friedman, 1994; Hollard and Delius, 1982; Parsons, 1987; Wohlschläger, 2001; Wohlschläger and Wohlschläger, 1998). Based on these findings, our initial prediction was that dogs would have been less accurate with wider rotations of stimuli, which would be consistent with a mental rotation process, as described in humans and in a sea lion (Mauck and Dehnhardt, 1997). However, our results did not support this hypothesis. Indeed, differently from what observed in humans, the different angles of stimulus rotation did not influence dogs’ accuracy.

Although all dogs were more accurate in discriminating upright stimuli than rotated stimuli, consistently with the performance observed in humans (Hollard and Delius, 1982), a sea lion (Stich et al., 2003), rhesus monkeys (Köhler et al., 2005), and a lion-tailed macaque (Burmann et al., 2005), we found no linear effect of the amplitude of stimulus rotation on performance. In addition, while all subjects performed significantly above chance level with upright stimuli, at the group level, their performance with rotated stimuli did not differ significantly from chance. When we looked at the effect of the degree of stimulus rotation, we found no differences for clockwise and counter-clockwise rotations of different angles (Figure 1). It is possible that a mental-rotation-like effect of stimulus rotation on generalization performance would appear only when testing a group of subjects who perform above chance level with rotated stimuli. However, also undergraduate students’ accuracy was not influenced by increasing angular disparity in a letter naming task (Risko et al., 2014), probably due to the simplicity of the task. Indeed, the students’ average mistake rate when reading blocks of 15 letters was 10.0% for upright letters, 11.7% for 45° rotated letters, and 8.8% for 90° rotated letters. Hence, it is possible that an effect of stimulus rotation on accuracy would be best observed for intermediate levels of task difficulty. This possibility should be addressed in further studies.

We also investigated sex and individual differences. We found no sex difference in the accuracy with rotated stimuli, while females were significantly more accurate than males during the test trials that presented the training (upright) shapes. We found large inter-individual variability in the number of sessions that dogs needed to learn the visual discrimination task. However, no sex or age differences emerged during training, meaning that females and males of all ages reached comparable levels of accuracy during the training phase, at similar speed. Previous research based on owners’ reports (e.g., Hsu and Serpell, 2003; Kubinyi et al., 2009; Serpell and Hsu, 2005), identified “trainability” as one of the factors explaining dogs’ inter-individual differences. The definition of trainability included both the willingness to obey to already acquired commands and the speed, distractability and resistance to correction when learning new tasks. According to this definition, none of these studies found conclusive sex differences over large samples. Based on our results, it is possible that the distinction between a training (when the behavior to be performed is not understood yet) and a test phase (when the behavior has already been acquired) should not be overlooked. Indeed, males and females might differ in their motivation to comply with tasks but not in their ability to learn them. Only the eight dogs that reliably acquired the visual discrimination task were tested on the rotated versions of the same stimuli.

The plots on individual performance (Figure 2) show that at least one female dog (dog 21) passed the generalization test consistently recognizing above chance level the reinforced stimulus even when this was rotated of all different angles, as indicated by the CIs for the fitted values being above 0.5. Because the model fitted to the data is blind to the performance being significantly above or below chance level, we also compared each individuals’ overall accuracy with rotated stimuli to chance level, using binomial tests. The results showed that one additional male dog (dog 22) performed significantly above chance level with rotated stimuli and one female (dog 29) performed significantly below chance level with rotated stimuli. Based on the performance of dogs 21 and 22, who selected the reinforced stimulus overall above chance level even when stimuli were rotated, we conclude that dogs have the (neuro-cognitive) potential to recognize rotated bidimensional objects. This result implies that dogs might have the capability to build a mental representation of the training stimuli, to remember it even when the stimuli are no longer present (during the test), and to compare it with the rotated point of view presented during test.

Interestingly, the two dogs performing above chance level were trained and tested on the same pair of stimuli (pair 2 in Figure 5), whereas other dogs (7 and 20) did not pass the generalization test with the same pair. On the other hand, the dog who performed significantly below chance level was trained and tested on the same stimuli (pair 3 in Figure 5) as the other two dogs (10 and 11) who tended to perform below chance level with counter-clockwise 90° rotations. The fact that dogs were misled by these specific stimuli being rotated of 90° can be explained in terms of local attention. The upright pair of stimuli is reported in the upper part of Figure S3. When this pair is rotated (lower part of Figure S3), a pattern present on the upright negative stimulus is recreated on the rotated reinforced stimulus. It is possible that dogs focused mainly on the highlighted part of the negative stimulus and consequently discarded the 90° rotated positive stimulus.

**Figure 4:**
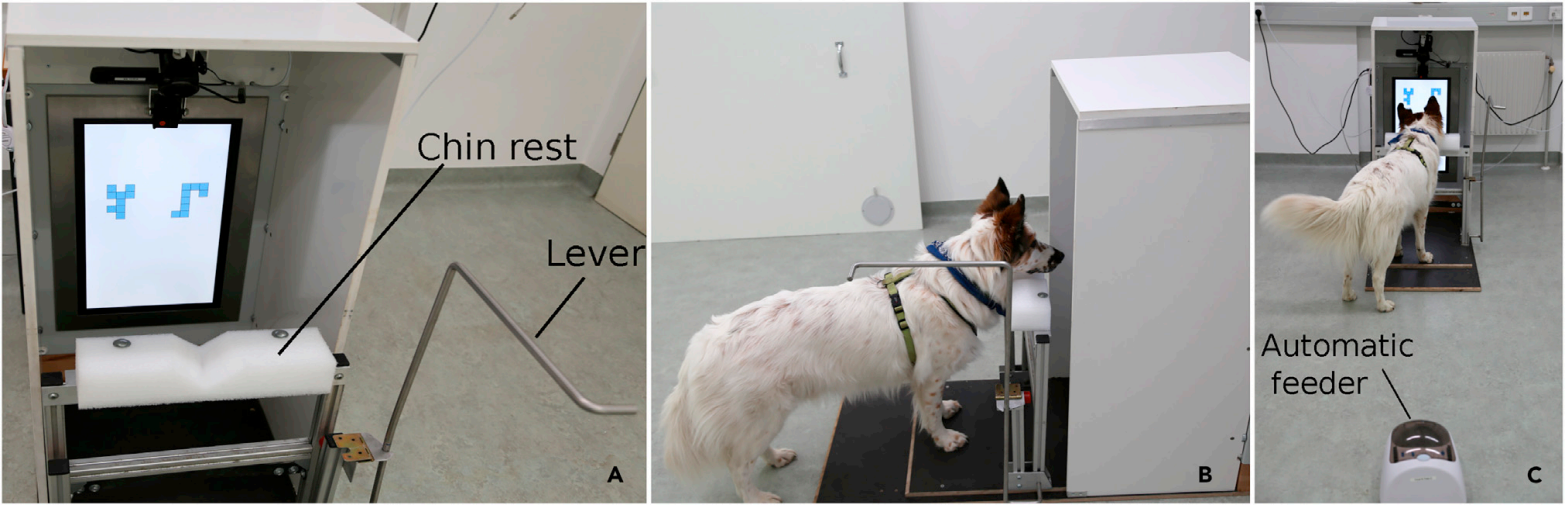
Experimental set-up (A) The chinrest, opposite to the touchscreen, was operated through the metallic lever on the right. Hence, the experimenter always stood on the dog's right side. (B) One of the subjects shows the starting position, watching the screen with head on the chinrest. (C) The automatic feeder behind the dog.

**Figure 5:**
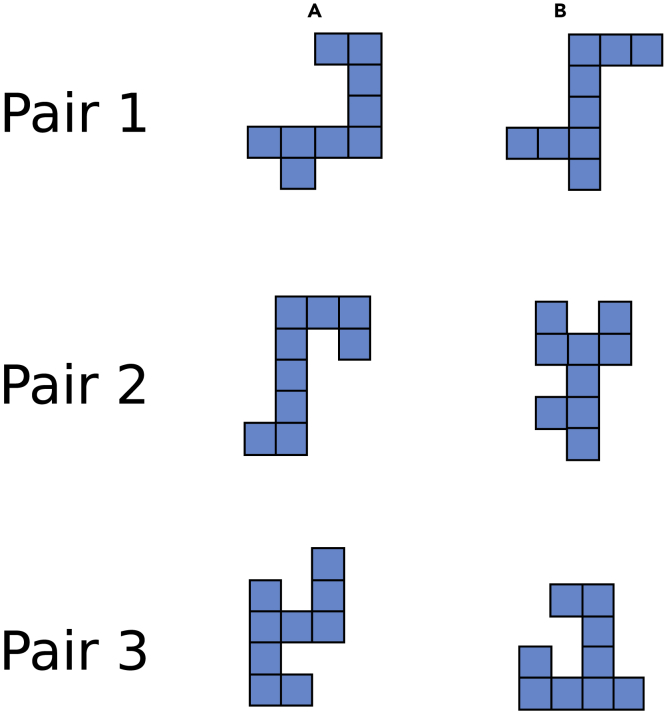
The three pairs of stimuli used in the whole experiment, portrayed in the upright orientation Each dog was randomly assigned to one of the pairs and, within the pair, to one reinforced stimulus.

Taken together, these results suggest that dogs might use individual strategies in solving this recognition of rotated objects task and that their performance was influenced by the specific stimuli, probably due to a local rather than a global focus of attention.

Individual differences in the strategies employed to solve a mental rotation task were already reported for three Rhesus monkeys (Köhler et al., 2005). Based on these, the authors speculated about the possible co-existence of two different mechanisms of visual processing (mental rotation and rotational invariance) within the same species. We currently cannot assess whether this variability characterizes other species as well due to the prevalence of single-case studies (e.g., Burmann et al., 2005; Stich et al., 2003) and group-level only analyses (e.g., Hollard and Delius, 1982; Hopkins et al., 1993; Shepard and Metzler, 1971).

Pitteri et al., 2014a tested dogs on the Navon task with compound stimuli (Navon, 1977) and found great inter-individual variability in global/local precedence. The authors concluded that the global/local strategy of processing in dogs is determined by the individuals’ life experiences in using visual information more than by a predisposition of the whole species. Hence, our eight dogs’ different life experiences until the beginning of the experiment might explain the inter-individual variability observed in the strategies to solve our task. Approximately two years later, Mongillo et al. (2017) re-tested the same dogs on the same task as Pitteri et al., 2014a and found a clearer global bias than in the original study. Instead, in a subsequent experiment, dogs were trained on a novel composite stimulus and, when tested, did not show an overall global precedence. In fact, they tended to invert their strategy relative to the original study. On average, despite being slightly higher than wide, our stimuli subtended a comparable area as those in these two studies.

We currently cannot pinpoint the strategy that dogs used to solve this task. In contrast to what is to be expected from the mental rotation and the object recognition accounts, dogs’ accuracy did not decrease systematically with increasing stimulus rotation. Moreover, dogs’ overall inaccuracy with rotated stimuli suggests that rotational invariance is unlikely to explain their performance. For better comparison with the existing literature, in a future experiment, it would be interesting to add mirror images discrimination to the task and to introduce three-dimensional perspective drawings of stimuli. Furthermore, a fundamental variable to measure in this kind of tasks is the reaction time. Indeed, previous studies with other species have mainly distinguished between rotational invariance and mental rotation processes based on reaction times. And even the well documented sex difference found in humans is mainly due to males being faster—though not more accurate—than females at all ages through the human lifespan (Linn and Petersen, 1985).

The amplitude and occurrence of head tilts in this setting were very low. Unlike previous studies (Risko et al., 2014; Sommese et al., 2021), we did not code head tilts dichotomously as present/absent but rather we measured how inclined dogs’ heads were before and after the stimulus onset. Indeed, we were interested in any head rotation around the z axis, even very small ones, because those might already facilitate the recognition of rotated objects. Moreover, while Risko et al. (2014) coded as head tilts only rotations larger than 10° and those that happened within 1 s from the stimulus onset, we coded the degrees of dogs’ head tilt within 3 s from stimulus onset, in each trial presenting rotated stimuli. We observed spontaneous head rotations wider than 10° on approximately 8% of the trials, while Risko et al. (2014) report that human participants, with unrestricted heads, exhibited this behavior on average in 16% of the trials of a letter naming task presenting rotated stimuli of 0°, ±45°, or ±90°. The limited movements we observed might be due to the chinrest inhibiting wider actions, apart from obvious anatomical differences between dogs and humans. In addition, the extreme difficulty of the task might explain the floor effect in the number of attempts dogs made to externally normalize the pictures. Indeed, if dogs did not recognize that the test stimuli consisted of rotations of the familiar (training) ones, they might have not realized that tilting their heads would decrease the cost of the mental task.

In our study, dogs did not exhibit head tilts in a preferred direction, but rather tended to tilt their heads in the direction in which the stimuli were rotated. Moreover, most of the observed head movements were characterized by small amplitude (average amplitude observed after stimulus onset: ca. 5°) and in general, they occurred at a low rate. Taken together, these elements let us speculate that the behaviors observed in this study in response to visual (rotated) stimuli and the head tilts observed by Sommese et al. (2021) in response to auditory stimuli might subtend different mechanisms and serve different functions. However, it is important to notice that in our controlled setting dogs could have not exhibited such wide head tilts (maximum head tilt observed after stimulus onset: ca. 26°) without lifting their head from the chinrest, a behavior they rarely exhibited, probably due to the previous training.

The limited and very brief head tilts we observed in the current study might be indicative of an attempt to ease the cognitive demands of the task, but they are also consistent with other interpretations. For example, the dogs might have been impatient to make a choice during the observation period and tilting their head could have constituted the first step in trying to overcome the chinrest that prevented them from moving forward. However, we found that the degrees of stimulus rotation had a significant effect on the wideness of head rotations measured after the stimulus onset. This means that, after the stimulus onset, dogs’ heads were more inclined for wider stimulus rotations and they were inclined in the same direction (clockwise or counter-clockwise) as the stimuli.

The wideness of dogs’ head tilts did not have an influence on accuracy in our task. The lack of difference in the amplitude of head tilts exhibited by females and males is consistent with the absence of sex differences in dogs’ accuracy with rotated stimuli. In Risko et al. (2014)’s experiments, no effect of head tilts was found on participants’ accuracy in a letter naming task, while both spontaneous and forced head tilts improved performance when participants had to read whole paragraphs rather than single letters. However, in Risko et al. (2014)’s third experiment, participants' heads were unrestrained, unlike our dogs’ heads. Moreover, owing to our relatively small sample size and the different level of task difficulty, it is possible that our study is underpowered to show the effect of head tilts on accuracy.

External normalization (e.g., head tilting) need not improve performance to be considered an instance of cognitive offloading. However, given that tilting the head did not improve dogs’ accuracy, it remains unclear whether such a behavior offloads on the body an internal computation (Risko et al., 2014). Hence, we conclude that our results provide no evidence for cognitive offloading in dogs.

In conclusion, although recognition of rotated two-dimensional shapes proved to be challenging in our setting, we showed that dogs have the potential to solve the task. We additionally showed that dogs’ head tilts could be systematically studied in a controlled setting; thus, we provided a methodology for studying cognitive offloading in non-human species. We did not find clear evidence that dogs tilted their heads as a means to offload a cognitive process onto their bodies. Future research should investigate whether non-human animals engage in cognitive offloading when facing moderately demanding tasks in this and in other domains, such as memory.

### Limitations of the study

In the present study, we chose to train dogs to lay their head on a chinrest before presenting them with visual stimuli. We chose to stabilize the dogs’ heads for different reasons. First, we aimed at ensuring that the dogs would observe the stimuli consistently across trials with regards to their body orientation and distance relative to the screen. Second, we wanted to minimize dogs’ impulsivity by forcing them to wait a pre-determined amount of time before allowing them to walk forward and touch the screen. Finally, the dogs’ head was required to remain in a pre-determined and consistent position in space to ensure precise coding of the video frames. The chinrest itself did not prevent dogs from tilting their head, as proven by the (small) tilts we observed. However, we cannot know if the chinrest training might have inhibited dogs’ willingness to tilt their head more widely, whether this effect might have been more pronounced for some individuals than others, and if wider head tilts would have influenced performance. Therefore, future studies assessing dogs’ head tilts should leave their heads unrestrained.

While previous studies have shown dogs are able to recognize 2D stimuli on the basis of visual cues alone (e.g., Müller et al., 2015; Pitteri et al., 2014b), it is likely that, under more ecological conditions, dogs do not need to rely exclusively on their visual modality to recognize previously encountered entities. Therefore, dogs’ scant performance with rotated stimuli might be due to a true difficulty of this species with recognizing familiar objects presented from a rotated perspective, or to the lack of ecological validity of the task and abstract nature of the stimuli. Future studies will need to assess which factors influence dogs’ performance (for example, biological relevance of the stimuli, ecological setting with cross-modal cues, and absence of postural constraints). While the visual discrimination between these particular shapes and the rotated object recognition tasks might have been too difficult for most of the dogs, in order to address the phenomenon of cognitive offloading, we had to confront subjects with a challenging task. If the task had been too simple, the dogs would have had no need to offload cognitive processing.

Finally, the limited sample size hinders the generalizability of our findings. In particular, the post-hoc speculation about a possible female advantage in performing an already acquired task but not in acquiring the task needs to be tested in future experiments across different tasks and with larger sample sizes.

## STAR★Methods

### Key resources table

**Table:** 

REAGENT or RESOURCE	SOURCE	IDENTIFIER
**Deposited data**		

Raw data	this study	Mendeley Data: https://doi.org/10.17632/khhkn6kcpm.1
Code for statistical analyses and plots	this study	Mendeley Data: https://doi.org/10.17632/khhkn6kcpm.1
R workspaces	this study	Mendeley Data: https://doi.org/10.17632/khhkn6kcpm.1

**Experimental models: Organisms/strains**		

Adult dogs (*Canis familiaris*)	private owners	“N/A”

**Software and algorithms**		

R version 3.6.3	R core team 2020	R: The R Project for Statistical Computing (r-project.org)
ImageJ	Schneider et al. (2012)	https://imagej.nih.gov/ij/

### Resource availability

#### Lead contact

Inquiries should be addressed to the lead contact, Lucrezia Lonardo (lucrezia.lonardo@vetmeduni.ac.at).

#### Materials availability

This study did not generate new unique materials

### Experimental model and subject details

#### Dogs (*Canis familiaris*)

All dogs who took part in this experiment were pets, brought to the lab by their volunteer owners. Prior to the beginning of the experiment, owners were informed about the aim and procedures of the study, and gave an informed written consent for their dogs. Breed, age, sex, previous touchscreen experience, number of training sessions and reinforced stimulus of each of the 38 dogs are reported in the supplemental information (Table S11).

Overall, 38 dogs (see Table S11) started in the experiment. Of these, five were excluded from the study at an early stage due to behavioural issues that were not compatible with learning or that posed a threat to the integrity of the experimental set-up (e.g., constant barking, excessive impulsivity, fear of the wooden apparatus). Four of these five dogs were excluded during the pre-training, the fifth after 3 training sessions. One additional dog passed away. A further nine subjects dropped out of the study at different stages due to limitations in owner availability. All other dogs completed at least 30 training sessions (600 trials) and were included in the analysis of this phase. All other dogs completed at least 30 training sessions (600 trials) and were included in the analysis of this phase. The resulting sample size comprised therefore 23 dogs trained over a total of 1157 sessions. Of these 23 dogs, only 8 met the learning criteria (see Paragraph “procedure” below) and were therefore tested with rotated stimuli.

The 8 tested dogs (4 females) had a mean age of 7 years (age range 3–9 years). Seven dogs had previously taken part in other experiments at the Clever Dog Lab. Table S12 summarises the main information regarding the 8 tested dogs. Dogs were randomly assigned to one of the three pairs of stimuli shown in Figure 5.

The study was discussed and approved by the ethics and animal welfare committee of the University of Veterinary Medicine of Vienna in accordance with GSP guidelines, national legislation and EU regulations.

### Method details

The experiment was conducted at the Clever Dog Lab, Messerli Research Institute, University of Veterinary Medicine (Vienna). Dogs were trained and tested in the same 6 × 3 m room, with the help of a semi-automated touchscreen and feeder. The automatic feeder used was a Premier Treat & Train, filled with dry food pellets. It was positioned 1.20 meter behind the dog (Figure 4C). The touchscreen was a Thin Film Transistor (TFT), with refresh rate of 60 Hz. It measured 46.5 × 27 cm (height x wideness) and it was inserted in a white wooden apparatus measuring 100 x 45.5 x 49 cm (height × depth × wideness). This minimised the possibility of human cueing and distractions for the dogs (Figure 4A). To approach the touchscreen, dogs had to walk on a black platform and lay their head on a chinrest (Figure 4B). The chinrest ensured a standardised position of the dogs′ head at the beginning of each trial (hence a rigorous video coding afterwards). The black platform measured 110 x 50 cm and the chinrest 9.2 × 32.7 (l × w). This was a rubber foam pillow with v-shaped indentation in the middle (depth: 2.2 cm). It was 5.5 cm deep on the sides. Dogs watched the stimuli on the screen over a distance of approximately 50 cm. The experimental set-up is portrayed in Figures 4 and 7.

**Figure 6:**
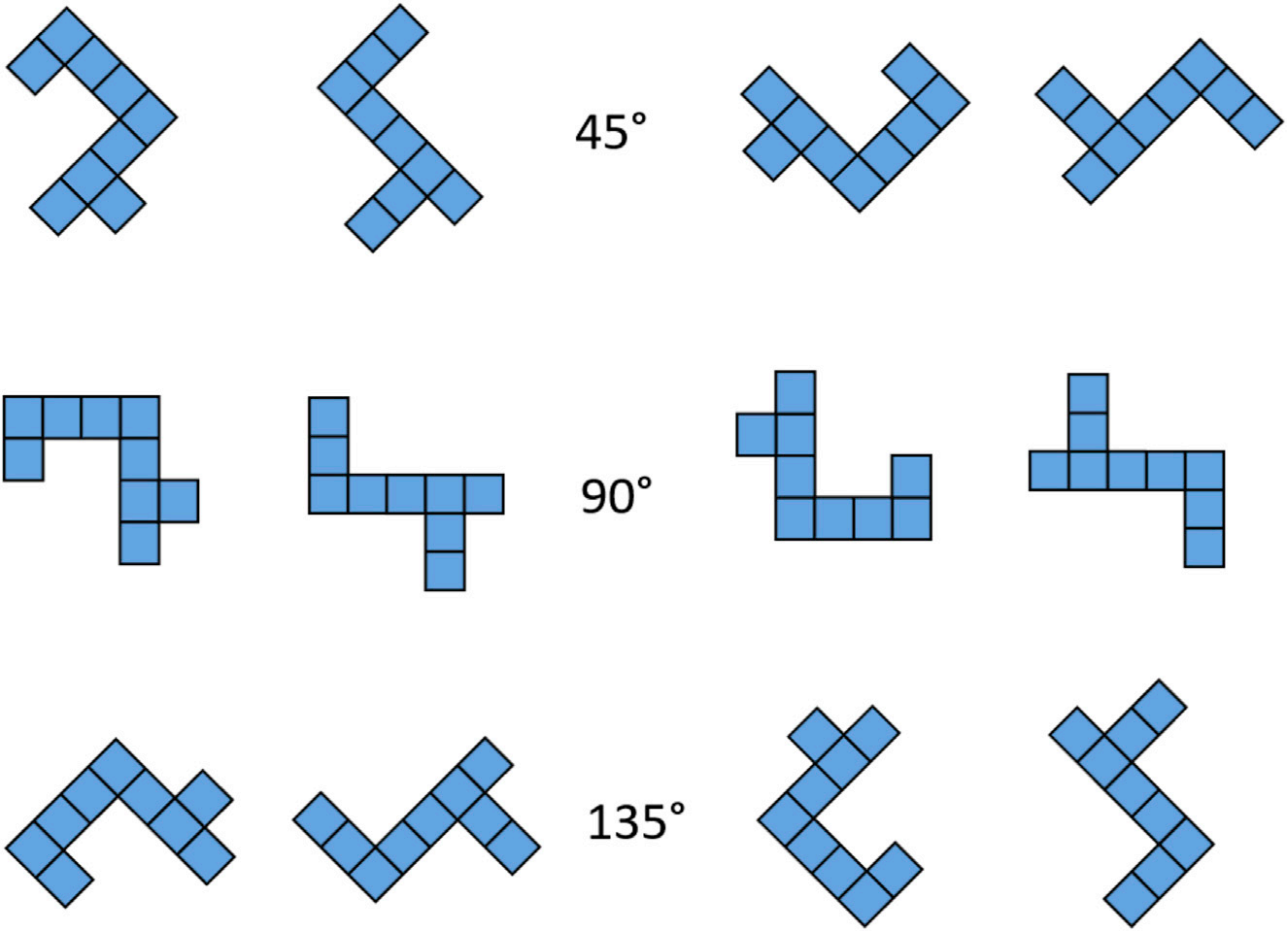
Examples of test stimuli Pair one is shown in all three rotated versions, rotated by 45° (top row), 90° (central row), and 135° (bottom row). Counter-clockwise rotations are shown on the left, clockwise rotations on the right.

**Figure 7:**
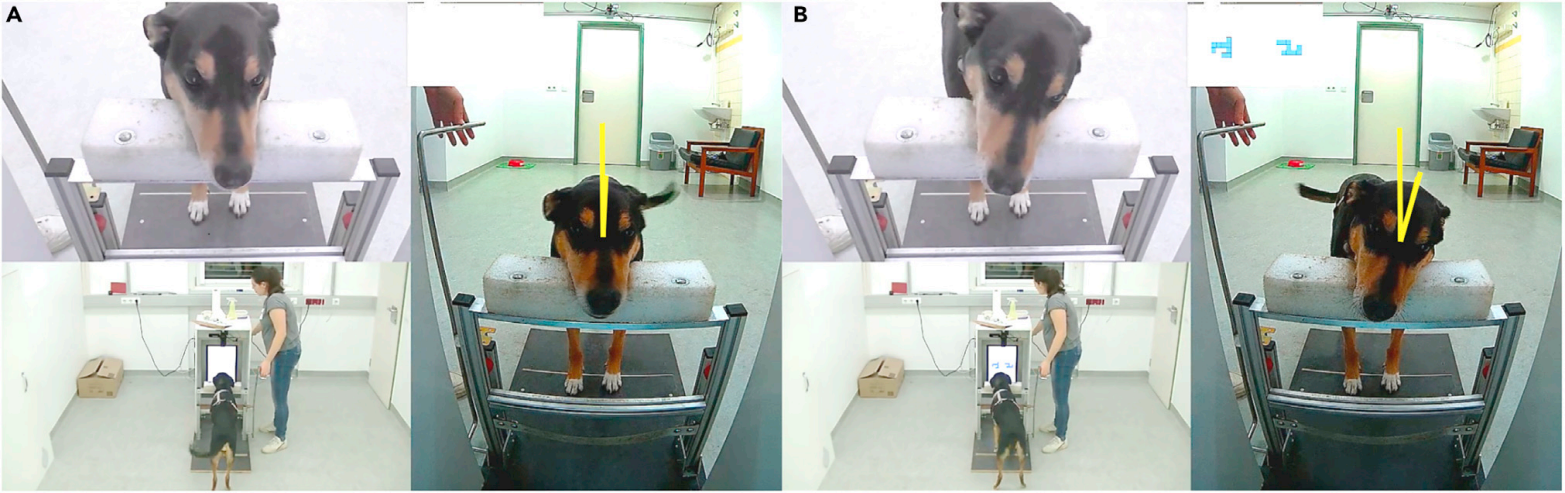
Examples of head tilt coding from video frames (A and B) Frame (A) and (B) come from the same test trial. Frame (A) is the one immediately preceding the stimulus onset, while frame (B) is the one containing the widest head tilt for that trial after the stimulus onset. The box in the upper part of both frames shows the content of the screen as viewed from the dog’s perspective. The yellow lines on the dog’s forehead show how the angles of head rotation were measured.

#### Stimuli

As stimuli, we adapted the two-dimensional Hollard and Delius (1982)’s shapes. We changed the colour of the stimuli to blue because this is one of the two hues falling in dogs’ visible spectrum (violet and blue-violet range: 430 to 475 nm wavelengths). Each stimulus was composed of nine adjacent squares forming an asymmetrical shape. The two shapes of each pair differed in the positioning of four of the squares. Each square measured 4 cm^2^ on the screen. We used three different pairs of stimuli to assess whether different shapes would have influenced dogs’ accuracy. All stimuli were between 8 to 10 cm high and between 6 to 10 cm wide. They subtended approximately between 9.2° to 11.4° (height) and between 6.9° to 11.4° (width) of visual angle. Variations in wideness and height depend on the specific stimulus considered. All the upright stimuli are reported in Figure 5 and a rotated pair is in Figure 6. Mirror invariance, the tendency to identify two mirror images as the same picture across left-right inversions, has been shown in humans and other primates (Biederman and Cooper, 1991; Logothetis et al., 1995; Pegado et al., 2014). Hence, we did not include mirror images in the task, as we wanted to increase the dogs’ possibilities of seeing a difference between the two alternatives.

#### Procedure

With one exception, the dog′s owner was present in the room throughout the whole experiment. To prevent interference, when present in the room, owners sat or stood more than 2 meters behind their dog. During test sessions, owners sat with their backs towards their dogs so that they could not see the screen, the experimenter nor the dog.

The experiment included three consecutive training phases (until dogs reached predefined learning criteria, outlined in the following Paragraph) and a subsequent test phase.

#### Training

We trained pet dogs to discriminate between the elements of a pair of geometrical shapes. Each dog was trained and tested on a single pair of stimuli. This means that, throughout the whole experiment, only one stimulus (S+) was associated to a food reward while the other (S-) never was. To avoid side biases, the left/right position of the S+ changed pseudo-randomly, having no more than three consecutive presentations of the correct stimulus on the same side.

Both training and test followed a simultaneous two-choice discrimination paradigm, previously used in touchscreen experiments for dogs (e.g., Müller et al., 2015). Dogs were trained to lay their heads on the chinrest and watch the screen prior to each trial. Once the dog was in this initial position, the experimenter (standing on the right side of the apparatus) presented the stimuli on the screen. After 3 seconds in which the dog watched the stimuli, the experimenter manually moved the chinrest to the floor using the lever shown in Figure 4A. Dogs were then free to step forward and touch the screen with their nose. After each touch, the stimuli disappeared. If the choice was correct, and the trial was a rewarded one, a tone and a food pellet were automatically emitted by the feeder behind the dog. Otherwise, the experimenter lifted the chinrest back in place and started a new trial when the dog was ready again. To shape this complex behaviour, training was sub-divided in the following stages:

1) Approach. Only for dogs with no or very little touch screen experience.

Dogs were trained to approach the touchscreen as soon as a stimulus (a large black dot) appeared on the white screen. They were encouraged with food to touch the black dot with their nose. Touch responses were rewarded with a dog food pellet that was automatically dispensed from the feeder behind the dog. Each session consisted of 20 trials. When dogs had reliably performed the approach-touch response in this phase, as judged by the experimenter, they moved on to the pre-training.

2) Pre-training: one shape - the reinforced stimulus. For every dog, at least 5 sessions.

Dogs needed to touch the only stimulus appearing on the screen (one of the blue upright shapes, Figure 5) to get a reward from the automatic feeder. At this stage, there was no possibility of doing wrong because if dogs touched the white screen, nothing happened while if they touched the shape, they got a reward. For each dog, the shape appearing on the screen in this phase was always the same and it was the reinforced stimulus for that dog. When dogs reliably mastered this phase, as judged by the experimenter, they moved on to the next one. Each session consisted of 20 trials.

3) Simultaneous two choice discrimination (100% rewarded)

Dogs needed to discriminate between two simultaneously presented stimuli, the correct stimulus S+ (the same as seen in pre-training) and the S–, which is a different blue training shape. The left/right position of the S+ changed pseudo-randomly with never more than 3 consecutive presentations of the same disposition. A reward was automatically dispensed for every correct response.

When dogs chose the S–, the correction procedure started: the stimuli would disappear and the screen would turn red until the experimenter pressed the forward button. The chinrest was lifted back in place so that the dog could start the new trial with the head in a standardised position. Each wrong choice was followed by a correction trial, presenting the same configuration of stimuli again. Correction trials were excluded from the analysis of performance.

Once a dog had reached the learning criterion (16 out of 20, i.e. 80% correct responses within a session in each of 3 consecutive sessions), it moved on to the next phase. If dogs found the discrimination too difficult, the experimenter could choose to insert a pre-training (phase 2) session (only one image) to recover the dog's motivation. In addition, the experimenter could decide to start a training day (phase 3) with a pre-training (phase 2) session.

In this crucial training phase, dogs needed to learn to be persistent with their choice. If a dog was stuck, the experimenter could cover (with her hand) the wrong alternative or she could point to the right stimulus to make the task obvious for the dogs.

4) Simultaneous two-choice discrimination training with partial reinforcement (85% rewarded)

In the last training phase, dogs needed to discriminate between two simultaneously presented stimuli, the correct stimulus S+ (the same seen in pre-training) and the S–, which was a different blue training shape. If dogs chose the S+, they had around 85% of chances of being rewarded.

On average 3 out of 20 trials in each session were not rewarded. During these unrewarded trials, nothing signalled the dog if they chose correctly or not. This partial reinforcement phase familiarised dogs with the reward contingency of the test sessions, in which 3 trials (those presenting the rotated stimuli) would have always been unrewarded.

Each dog was trained until they reached the learning criterion of 80% correct responses in 3 consecutive sessions (48 correct/60 trials) or for at least 30 sessions without reaching this criterion. Some of the dogs were trained for longer as the owners were available to continue with the training. Only eight out of 23 dogs reached this learning criterion and moved on to the test phase.

#### Test

Each test day started with a training session for rehearsal purposes. Then we conducted the test sessions, consisting of 20 trials each. Within these 20 trials, 17 presented the familiar training (upright) stimuli and 3 presented novel stimuli, which were the rotated versions of both the S+ and the S–. With this schedule we prevented dogs from losing motivation during the test sessions, as rotated stimuli were never followed by a reward.

The left-right position of the two stimuli on the screen was pseudo-randomly balanced.

During each test session, the 3 trials presenting rotated stimuli (test trials) were pseudo-randomly interspersed among trials presenting upright stimuli (training trials), with a test trial always followed at least by one training trial. Furthermore, the first trial in a test session was always a training trial.

The transitional probabilities from one angle of rotation to another were balanced across the 24 test sessions.

### Quantification and statistical analyses

The touchscreen automatically scored whether the response was correct or not for each trial.

For each dog, we looked at the individual learning curve by plotting the accuracy for every session over the course of all training sessions (Figure S1).

A camera mounted on the top of the screen recorded the dogs’ head while subjects were watching the screen with the head positioned centrally and straight on the chinrest Figure 4). Therefore, we were able to code precisely whether and how much dogs inclined their heads while watching the rotated stimuli. We extracted 180 frames (60 per second) from the test videos. The time window we considered went from the moment before the stimulus onset until the experimenter′s first movement to lower the chinrest (approximately 3 seconds, during which dogs were observing the screen with their head on the chinrest). We visually inspected the frames to determine the one with maximum head tilt (after stimulus onset). We measured the angle of head tilt as the angle between the dogs′ forehead-middle of the eyes line and the vertical axis (angles drawn in yellow in Figure 7) by using the angle tool of the software ImageJ (Schneider et al., 2012).

We calculated the difference between the head tilt visible in the frame with widest rotation after the stimulus onset and the head tilt in the frame immediately preceding the stimulus onset. To assess the agreement between two coders on this variable and on the degrees of head tilts after the stimuli onset, we calculated Intra-class Correlation Coefficients on a subset of 60 observations of different subjects. In detail, we used a two-way random-effects model to assess absolute agreement between the two coders, one of which was blind to the experimental hypothesis and conditions. Because we were interested in the reliability of our dependent variables (as coded by a single rater) we calculated the ICC on single measurements (ICC type: “single”). The resulting inter-raters reliability was acceptable (for both variables, ICC: 78%; N = 60, p < 0.001).

To validate the reliability of the automatic scoring made by the touchscreen, a second coder also scored the dogs’ accuracy from video recordings. We calculated an unweighted Cohen’s Kappa on 60 observations of different subjects. The agreement between touchscreen and second rater was almost perfect (Kappa = 0.966, N = 60, p < 0.001).

To analyse dogs’ performance during training (phases 3 and 4), we fitted a generalized linear mixed model (GLMM; Baayen, 2008) with binomial error structure and logit link function (McCullagh and Nelder, 1989). We modelled the proportion of correct responses per session as a function of sex and its interaction with session number (fixed effects). In addition to the test predictors, we included the session number, age and age squared and also the interactions between age and age squared, on the one hand, and session number, on the other, as control predictors with fixed effect. We included the interaction between sex and session number because a sex difference could manifest in one sex acquiring the capability of performing successfully faster than the other. We included age squared because, given the wide age range, we hypothesised that middle-aged dogs might have learned the task faster (i.e., they might have reached higher levels of accuracy within fewer sessions). We included dog identity, reinforced stimulus and session ID (nested within dog, as all subjects were trained for months) as random intercept effects. Finally, in this and in all the following models, all theoretically identifiable random slopes were included. This ensured that type-I error rate was kept at the nominal level of 0.05 and avoided overconfidence in the precision of the fixed effects estimates (Barr et al., 2013; Schielzeth and Forstmeier, 2009). Namely, in this model we included: the random slope of session number within dog and that of sex, of age and of the interaction between age squared and session number within reinforced stimulus.

We checked the distribution of the random effects and we verified that the model was not overdispersed. The result showed no issue in this regard (dispersion parameter = 1.004).

To test the significance of the main effect of sex and its possible interaction with session number, we used a likelihood ratio test (Dobson, 2002). This compared the fit of the full model with that of a null model, lacking the fixed effects of sex and its interaction with session number but retaining the same random effects structure and all other fixed effects present in the full model (Schielzeth and Forstmeier, 2009). For this and the following models, to draw inference about the individual predictors, we always used the function drop1 (Chambers and Hastie, 1992), which drops each fixed effect from the model (one at a time) and uses a likelihood ratio test to compare the full with the respective reduced models (Barr et al., 2013).

We assessed model stability with regards to the estimated coefficients and standard deviations by excluding the levels of the random effects one at a time (Nieuwenhuis et al., 2012). This revealed the model to be of good stability.

We fitted the model in R (version 3.6.3, R Core Team 2020) using the function glmer of the package lme4 (version 1.1-21; Bates et al., 2015).

A second GLMM with the same error structure and link function was fitted to analyse dogs’ accuracy during test. We modelled the proportion of correct responses as a function of the angle of rotation of the stimuli and sex (fixed effects). In addition to the test predictors, we included direction of rotation (factor with levels: none, clockwise, and counter-clockwise), session number and age as control predictors with fixed effect. We included dog identity, reinforced stimulus, and session ID (nested within dog) as random intercept effects. The latter allowed the possibility of variation among sessions within dogs as all dogs were tested across several weeks. Finally, all theoretically identifiable random slopes were included in the model. Namely, these were the random slope of direction of rotation, stimulus rotation and session number within dog; direction of rotation, stimulus rotation, session number, sex and age within reinforced stimulus; and, finally, direction of rotation and stimulus rotation within session ID.

Initially we fitted a maximal model (Barr et al., 2013), including also the correlations among random intercepts and slopes. However, all absolute correlation parameters were close to 1 and hence not identifiable (Matuschek et al., 2017). Therefore, we fitted a second model excluding the correlations. We assessed the capability of the two models to fit the data comparing their log-likelihoods (−1545.569 (df = 60) for the maximal model; −1559.429 (df = 23) for the model without the correlations). As removing the correlation parameters only led to a moderate decrease in model fit, we used the model without correlations for further analysis.

Prior to fitting the model, we z-transformed rotation, session number and age to a mean of zero and a standard deviation of one to increase the likelihood of the model to converge. We manually dummy coded and centred the factors direction of rotation (levels: no rotation, clockwise rotation and counterclockwise rotation; no rotation was set as reference category) and sex before including them as random slopes.

We assessed model stability with regards to the estimated coefficients and standard deviations by excluding the levels of the random effects one at a time (Nieuwenhuis et al., 2012). This revealed the model to be of good stability. We bootstrapped model estimates using the function bootMer of the package lme4 (Bates et al., 2015). We conducted an additional bootstrap, conditioning in the particular levels of the random effects (setting the argument use.u to “true”) which allowed to infer about the performance of the individual dogs.

We fitted the model in R (version 3.6.3, R Core Team 2020) using the function glmer of the package lme4 (version 1.1-21; Bates et al., 2015).

The sample for this model comprised 3840 observations, 576 of which were trials with rotated stimuli. Each one of the 8 dogs contributed equally to the number of observations (480 trials, obtained over 24 test sessions for each participant). The total number of incorrect choices was 657 while correct choices were 3183.

Because the results of this model suggested an effect of session number on accuracy, we further analysed only the performance with rotated stimuli (that were never reinforced) to test whether the effect of session number was driven by the reinforced trials with upright stimuli. To run this post-hoc manipulation check, we fitted a second model, identical to the first one but comprising only the 576 trials with rotated stimuli.

We additionally compared each individual’s overall performance with rotated stimuli to chance level. To avoid multiple testing and the consequent risk of increasing the likelihood of type I error, we did not run a significance test for each dog and angle of stimulus rotation. Instead, to infer about individual performance, we used the confidence intervals for the fitted values shown in Figure 2. Confidence intervals not comprising the value of 0.5 are indicative of performance significantly above chance level.

Finally, to quantify the relative contribution of the reinforced stimulus on accuracy, we compared the estimate of stimulus rotation (fixed effect) to the estimated standard deviations within reinforced stimulus (random intercept).

We modelled the degrees of head tilt after the stimulus onset as a function of the amplitude of stimulus rotation and sex using a linear mixed model (Baayen, 2008) with Gaussian error distribution. For both stimulus and head rotations, we transformed counter clockwise rotations to negative numbers. Prior to fitting the model, we z-transformed stimulus rotation (45°, 90° and 135°) to a mean of 0 and a standard deviation of 1. As we did not find any effect of direction of stimulus rotation on accuracy (paragraph “results - accuracy during test”), we did not include the direction of stimulus rotation (clockwise vs. counter clockwise) in this model. We included dog identity and reinforced stimulus as random intercept effects. Finally, all theoretically identifiable random slopes (stimuli rotation within subject and within reinforced stimulus) were included in the model. We fitted the model in R (version 3.6.3, R Core Team 2020) using the function lmer of the package lme4 (version 1.1-21; Bates et al., 2015).

Additionally, we run a simple regression (general linear model with Gaussian error structure and identity link) for each dog, to investigate also at individual level the effect of the angle of stimulus rotation on wideness of the head tilts exhibited after the stimulus onset. As described for the model above, we transformed counter clockwise rotations to negative numbers and z-transformed the angles of stimulus rotation. To fit the models, we used the function lm in R.

We checked the homogeneity and normality of the distribution of the residuals by inspecting qqplots. Model stability was evaluated through standardised DFFit-values (which compare the fitted values of a model using all data with those of a model with cases excluded one at a time) and Cook’s distance, a measure of the influence of each data point on model estimates (Queen and Keough, 2002)

To analyse the influence of head tilt on performance, we fitted a generalized linear mixed model (GLMM; Baayen, 2008) with binomial error structure and logit link function (McCullagh and Nelder, 1989). We modelled the proportion of correct responses as a function of the absolute difference between the degrees of head tilt after the stimulus onset and the degrees of head tilt before the stimulus onset. The larger this absolute angle, the more accurate we expected dogs to be. We additionally included sex as test predictor and dog identity and reinforced stimulus as random intercept effects. Finally, as in the previous models, all theoretically identifiable random slopes (absolute angle of head rotation within subject and within reinforced stimulus) were included.

To evaluate the main effect of head tilts and sex on accuracy we compared the fit of the full model with that of a null model lacking these two effects in the fixed effects part using a likelihood ratio test (Dobson, 2002).

For 79 trials it was not possible to measure an angle of head rotation before stimulus onset due to experimenter’s mistake in the procedure. For one trial it was not possible to measure the head rotation after the stimulus onset and whether the choice was correct or not due to touchscreen malfunctioning. Hence, the sample size for both models described in this section comprised 508 trials, 252 for counter clockwise-rotated stimuli and 256 for clockwise rotations measured from 8 subjects (between 28 and 78 trials per subject) and 5 different reinforced stimuli (between 28 and 78 trials per reinforced stimulus). In detail, for dogs 10 and 22, the model included 37 and 28 complete observations respectively. For dog 21, 68 complete observations; for dog 07, 76 complete observations, for dog 37, 71 complete observations and for dog 29, 72 complete observations. Dogs 07, 11 and 20 were tested on 2 additional sessions (6 trials with rotated stimuli) relative to the other dogs. For dogs 11 and 20 all the 78 observations were available.

For both models, we assessed stability by excluding the levels of the random effects one at a time (Nieuwenhuis et al., 2012) and calculated 95% confidence intervals conducting a parametric bootstrap based on 1000 repetitions. Individual effects were tested using likelihood ratio tests.

## Data Availability

• The raw datasets have been deposited and are currently available at Mendeley Data: http://dx.doi.org/10.17632/khhkn6kcpm.1 The DOI is listed in the key resources table.• The R code used to analyse and plot the data has been deposited and is currently available at Mendeley Data: http://dx.doi.org/10.17632/khhkn6kcpm.1 The folder “Training and learning curves” contains the scripts and data files for Figure S1 and Tables S1–S4. The folder “Recognition of rotated objects_accuracy during test” contains the script, workspace and data file for Figures 1 and 2 and for Tables S5 and S6. The folder “Cognitive offloading_head tilts” contains the script, workspace and data file for Figures 3 and S2 and Tables S7–S10. The folder “R functions” contains the custom R functions used for model stability, diagnostics and confidence intervals. The DOI is listed in the key resources table.• Any additional information required to reanalyse the data reported in this paper is available from the lead contact upon request.
