# Supplementary material for: Recognition of rotated objects and cognitive offloading in dogs
Source: iScience. 2022 Jan 26;25(2):103820. doi: 10.1016/j.isci.2022.103820 (PMC8841888; doi:10.1016/j.isci.2022.103820)
Supplement: Document S1. Figures S1–S3 and Tables S1–S12 [file mmc1.pdf]

iScience, Volume *25*

Supplemental information

Recognition of rotated objects and cognitive offloading in dogs

Lucrezia Lonardo, Elisabetta Versace, and Ludwig Huber

Supplemental figures

Learning curves

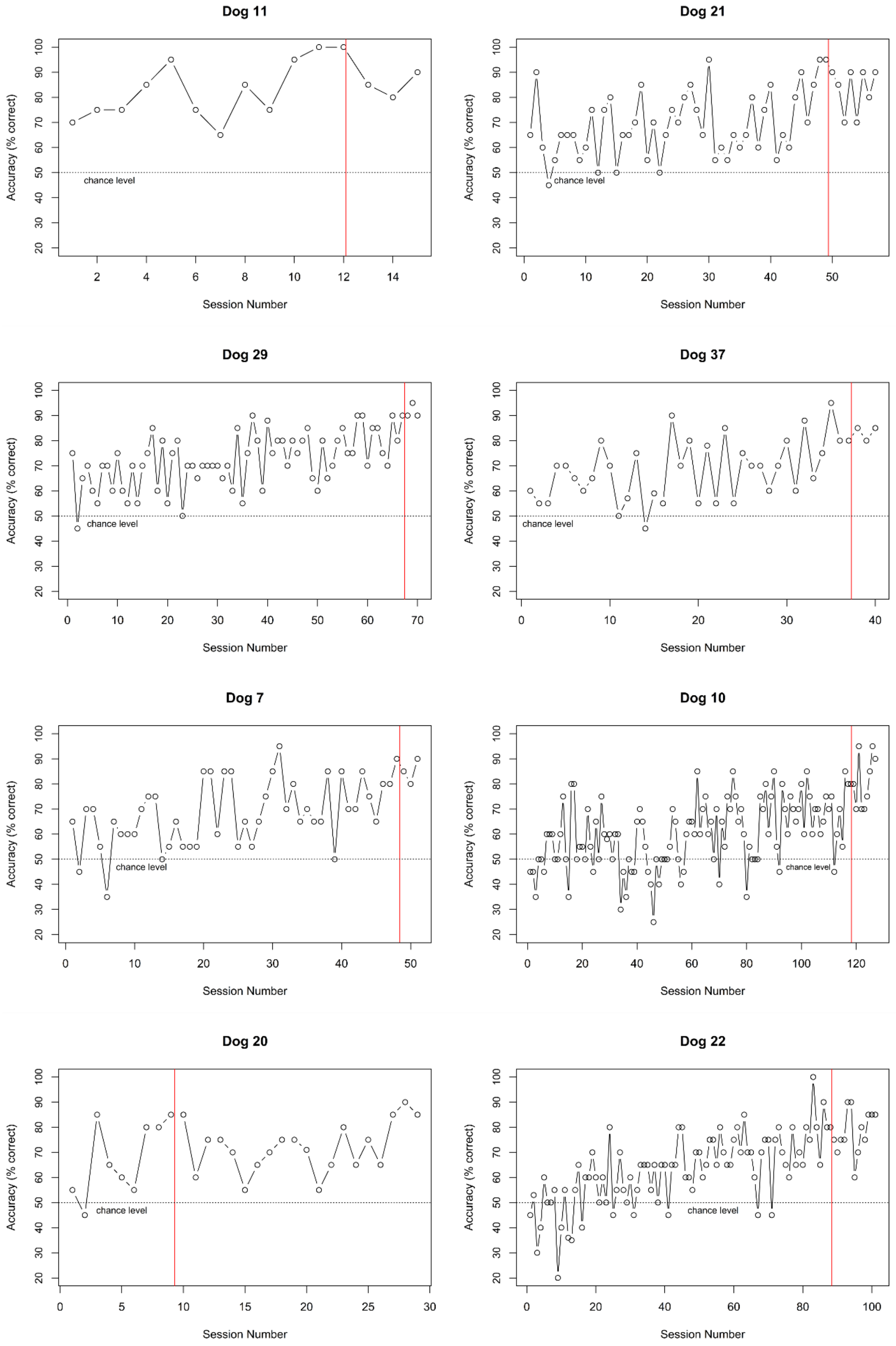

Figure S1 – related to “STAR methods/Quantification and statistical analyses”. The 8 tested dogs´ learning curves: percentage of correct responses in each session against number of training sessions. The plots show the performance during training phases 3 and 4. The vertical line indicates the passage from training phase 3 to 4 (partially reinforced). The horizontal dashed line indicates chance level (50%).

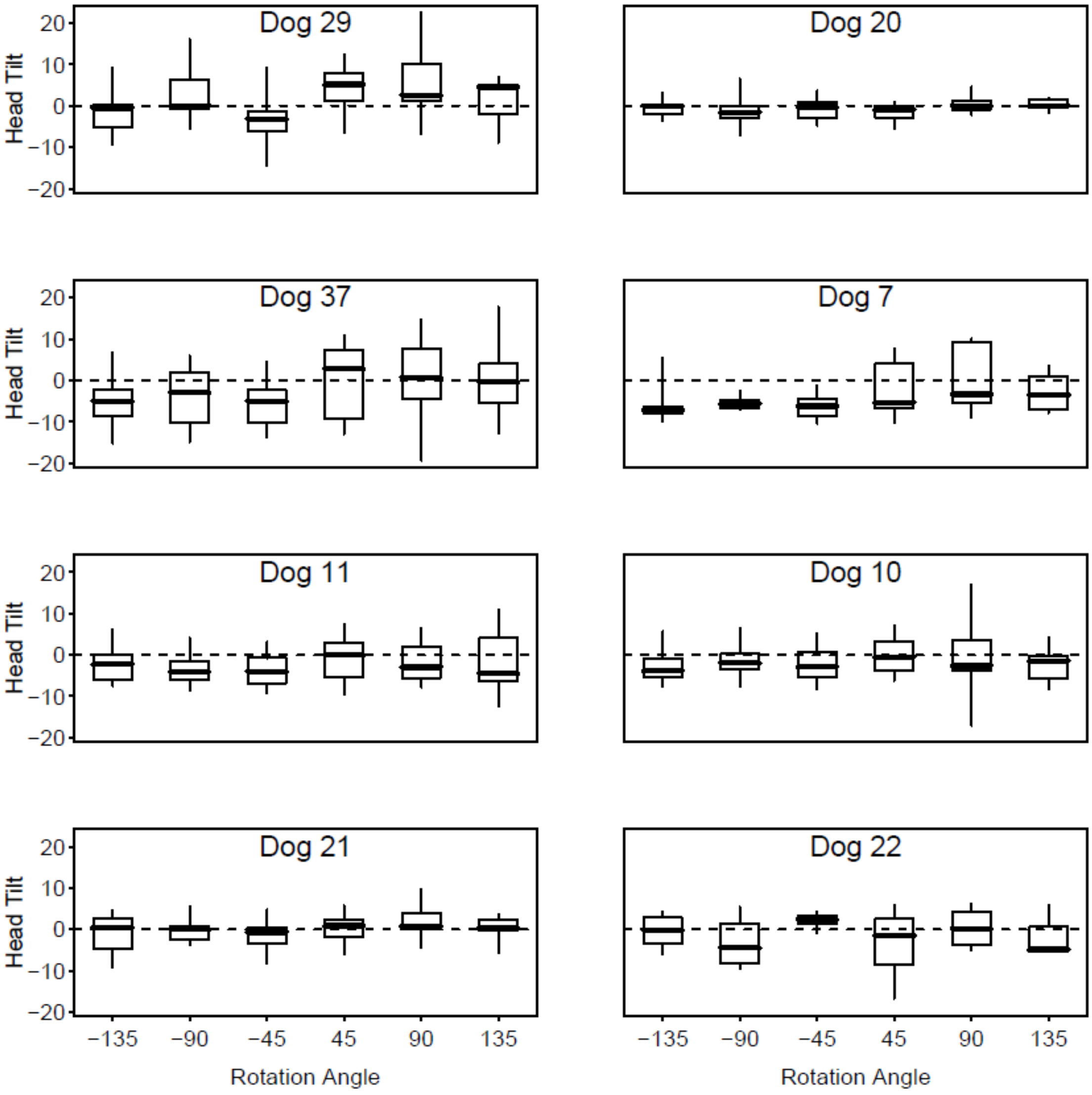

Figure S2 – Related to Figure 3. Each individual's head tilts after stimuli onset as a function of stimuli rotation. On both axes, negative numbers refer to counter clockwise rotations and positive numbers to clockwise rotations. The y-axis shows the degrees of dogs' head tilts after the stimuli onset.

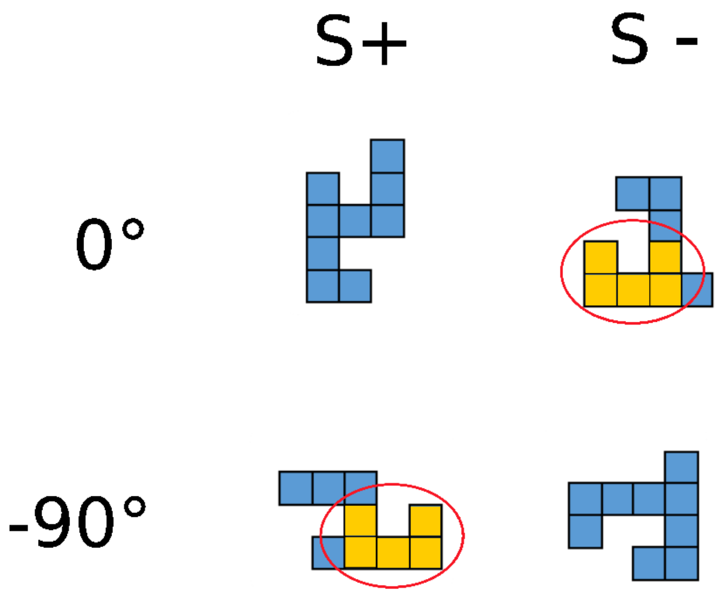

Figure S3 – related to Figures 5 and 6. Stimulus pair 3 (Figure 5) is shown upright (0° of rotation, upper part of the figure) and counter clockwise rotated of 90° (lower part of the figure). In both cases, the reinforced stimulus (S+) is shown on the left and the negative stimulus (S -) on the right. If dogs focused their attention on local details of the contours, they might have been misled for example by the appearance, on the rotated S+, of a pattern they learned to avoid on the upright S-.

Supplemental tables

Accuracy during training

*Table S1. Training performance. Accuracy (mean % correct responses) during training phases 3 and 4 for the 8 dogs that reached the learning criteria. Related to "STAR methods/*Quantification and statistical analyses*"*

Dog	% Correct	Training trials before test
**07**	69%	1020
**10**	61%	2515
**11**	83%	300
**20**	71%	567
**21**	71%	1140
**22**	65%	2002
**29**	73%	1396
**37**	69%	760

*Table S2. Accuracy during training. Results of the full model, with the interactions. Related to "STAR methods/*Quantification and statistical analyses*".*

	Estimate	Std. error	upper CI	lower CI	χ^2	P	Min	Max
**Intercept**	1.030	0.385	0.314	1.725	(1)	(1)	0.616	1.373
**Age (2)**	-0.019	0.148	-0.251	0.227			-0.166	0.354
**Session number (3)**	0.442	0.170	0.187	0.704			0.315	0.627

**Sex (4)**	-0.213	0.271	-0.649	0.221			-0.518	-0.067
**Age²**	-0.327	0.227	-0.725	0.107			-0.488	0.044
**Sex*session number**	0.034	0.158	-0.208	0.280	0.048	0.827	-0.115	0.108
**Age*session number**	-0.021	0.081	-0.155	0.127	0.062	0.804	-0.088	0.206
**Age²*session number**	-0.145	0.090	-0.311	0.033	1.741	0.187	-0.206	0.071

(1) Not indicated due to its very limited interpretation

(2) z-transformed to a mean of 0 and a standard deviation of 1. Original mean and standard deviation were 7.134 and 3.131 respectively.

(3) z-transformed to a mean of 0 and a standard deviation of 1. Original mean and standard deviation were 31.126 and 24.587

(4) for the random effects part: dummy coded with "female" being the reference category

Columns "min" and "max" show the minimum and maximum of the estimates obtained after case-wise deletion of the levels of the random effects (model stability).

*Table S3. Accuracy during training. Results of the first reduced model, lacking the non-significant interactions between sex and age² and session number. Related to "STAR methods/*Quantification and statistical analyses*".*

	**Estimate**	**Std. Error**	**upper CI**	**lower CI**	**χ²**	**P**
**Intercept**	0.949	0.338	0.344	1.561	(1)	(1)
**Age (2)**	0.027	0.145	-0.200	0.233		
**Session number (3)**	0.325	0.116	0.118	0.515		
**Sex (4)**	-0.371	0.233	-0.754	0.009	1.918	0.166
**Age²**	-0.092	0.226	-0.442	0.283	0.166	0.684
**Age*session number**	0.000	0.059	-0.124	0.126	0.000	0.994

(1) Not indicated due to its very limited interpretation

(2) z-transformed to a mean of 0 and a standard deviation of 1. Original mean and standard deviation were 7.134 and 3.131 respectively.

(3) z-transformed to a mean of 0 and a standard deviation of 1. Original mean and standard deviation were 31.126 and 24.587

(4) for the random effects part: dummy coded with "female" being the reference category

*Table S4. Accuracy during training. Results of the second reduced model, additionally lacking the interaction between age and session number. Related to "STAR methods/*Quantification and statistical analyses*".*

	**Estimate**	**Std. Error**	**upper CI**	**lower CI**	**χ²**	**P**

**Intercept**	0.950	0.333	0.323	1.582	(1)	(1)
**Age (2)**	0.027	0.117	-0.150	0.197	0.293	0.588
**Session number (3)**	0.326	0.116	0.134	0.523	6.843	0.009
**Sex (4)**	-0.372	0.233	-0.734	0.031	4.684	0.030
**Age^2**	-0.092	0.224	-0.470	0.230	0.001	0.979

(1) Not indicated due to its very limited interpretation

(2) z-transformed to a mean of 0 and a standard deviation of 1. Original mean and standard deviation were 7.134 and 3.131 respectively.

(3) z-transformed to a mean of 0 and a standard deviation of 1. Original mean and standard deviation were 31.126 and 24.587

(4) dummy coded with “female” being the reference category

Accuracy during test

Table S5. Test performance. Each dog's accuracy during test with upright and rotated stimuli. Related to Figures 1 and 2.

Dog	% Correct with upright stimuli	% Correct with rotated stimuli	comparison with chance level – P value
**07**	79%	60%	0.125
**10**	84%	43%	0.289
**11**	92%	51%	0.906
**20**	87%	54%	0.556
**21**	92%	72%	p<0.001*
**22**	87%	68%	0.003*
**29**	92%	35%	0.013*
**37**	90%	56%	0.410

Each dog was tested on 408 trials with upright stimuli and on 72 trials with rotated stimuli. Trials with rotated stimuli were fully balanced with regards to direction of rotation (clockwise and counter clockwise) and angle of rotation (45, 90 and 135 degrees) across individuals. Each dog's overall performance with rotated stimuli was compared to chance level (50% accuracy) using two-tailed binomial tests.

Table S6. Accuracy during test. Results of the model investigating the effect of stimuli rotation on performance. Related to Figures 1 and 2.

Term	Estimate	SE	lower CI	upper CI	χ^2	DF	P	min	max
Intercept	2.362	0.099	2.175	2.579			(1)	2.289	2.511
Rotation (2)	0.021	0.095	-0.171	0.214	0.048	1	0.826	-0.070	0.186
Rotation direction ccw (3)	-2.021	0.390	-2.787	-1.266	17.127	2	<0.001	-2.643	-1.577
Rotation direction cw (3)	-1.885	0.328	-2.558	-1.213				-2.463	-1.409
Session number (4)	0.300	0.050	0.208	0.395	13.106	1	<0.001	0.270	0.327
Sex (5)	-0.599	0.137	-0.895	-0.313	6.943	1	0.008	-0.753	-0.423
Age (6)	-0.029	0.062	-0.142	0.080	0.213	1	0.645	-0.115	0.025

(1) Not indicated due to its very limited interpretation

(2) z-transformed to a mean of 0 and a standard deviation of 1. The mean and standard deviation of the original variable were 13.5 and 35.151 respectively

(3) Dummy coded with "no rotation" being the reference category. Ccw: counter clockwise; cw: clockwise; the indicated test refers to the overall effect of the factor

(4) z-transformed to a mean of 0 and a standard deviation of 1. The mean and standard deviation of the original variable were 12.5 and 6.923 respectively

(5) Dummy coded with "female" being the reference category

(6) z-transformed to a mean of 0 and a standard deviation of 1. The mean and standard deviation of the original variable were 7 and 1.854 respectively

Head tilts and their influence on performance

Table S7. Head tilts after the stimulus onset. Results of the model investigating the effect of stimuli rotation and sex on wideness of head tilts measured after the stimulus onset. Related to Figure 3.

	Estimate	SE	Upper CI	Lower CI	χ^2	P	Min	Max
**Intercept**	-0.84	0.84	-2.39	0.86		(1)	-1.11	-0.52
**Stimulus rotation (2)**	0.97	0.35	0.34	1.64	5.48	0.019*	0.64	1.17
**Sex (3)**	-0.59	1.08	-2.70	1.53	0.29	0.589	-1.34	0.51

(1) Not indicated due to its very limited interpretation

(2) z-transformed to a mean of 0 and a standard deviation of 1. Original mean and standard deviation were 89.56 and 36.90 respectively.

(3) dummy coded with “female” being the reference category

As above, Min and Max refer to the model stability.

*Table S8. Difference in head tilt before and after stimulus onset. Results of the model investigating the effect of stimuli rotation and sex on wideness of head tilts measured as the difference between the head rotation after and before stimuli onset. Related to “STAR methods/*Quantification and statistical analyses*”.*

	Estimate	SE	Upper CI	Lower CI	χ^2	P	Min	Max
**Intercept**	0.591	0.725	-0.826	2.027		(1)	-0.098	1.233
**Stimulus rotation (2)**	-0.216	0.176	-0.555	0.135	1.512	0.219	-0.322	-0.137
**Sex (3)**	-0.038	0.902	-1.903	1.826	0.001	0.973	-0.644	0.957

(1) Not indicated due to its very limited interpretation

(2) z-transformed to a mean of 0 and a standard deviation of 1. Original mean and standard deviation were 89.56 and 36.90 respectively.

(3) dummy coded with “female” being the reference category

*Table S9. Results of the model investigating the effect of head tilts and sex on accuracy. Related to STAR methods/*Quantification and statistical analyses*”.*

	Estimate	SE	Upper CI	Lower CI	χ^2	P	Min	Max
**Intercept**	0.19	0.24	-0.28	0.66		(1)	-0.09	0.42
**Head tilt (2)**	0.06	0.10	-0.16	0.28	0.36	0.546	0.02	0.13
**Sex (3)**	0.17	0.35	-0.50	0.85	0.26	0.607	-0.12	0.53

(1) Not indicated due to its very limited interpretation

(2) Absolute value of the difference between head rotation after and before SO. Z-transformed to a mean of 0 and a standard deviation of 1. Original mean and standard deviation were 2.92 and 3.00, respectively.

(3) dummy coded with "female" being the reference category

Table S10. Results of the simple regressions investigating, for each dog, the effect of the angle of stimulus rotation on the degrees of head tilt measured after the stimulus onset. Related to Figure 3 and Figure S2.

	Estimate	SE	Upper CI	Lower CI	t	P	Min	Max
**Intercept Dog 7**	-2.273	0.600	-3.468	-1.077	- 3.788	<0.001	-2.443	-2.082
**Angle of stimuli rotation Dog 7 (1)**	0.529	0.599	-0.664	1.723	0.884	0.38	0.293	0.795
**Intercept Dog 10**	-3.696	0.946	-5.617	-1.775	-3.906	<0.001	-3.908	-3.302
**Angle of stimuli rotation Dog 10 (1)**	1.709	0.996	-0.314	3.731	1.715	0.095	1.109	2.053
**Intercept Dog 11**	-0.451	0.292	-1.032	0.129	-1.548	0.126	-0.533	-0.337
**Angle of stimuli rotation Dog 11 (1)**	0.394	0.291	-0.185	0.973	1.354	0.180	0.287	0.470
**Intercept Dog 20**	1.607	0.807	0.000	3.214	1.991	0.050	1.385	1.916
**Angle of stimuli rotation Dog 20 (1)**	1.487	0.805	-0.115	3.090	1.849	0.068	1.238	1.771
**Intercept Dog 21**	-1.638	0.687	-3.010	-0.266	-2.384	0.020	-1.924	-1.309
**Angle of stimuli rotation Dog 21 (1)**	0.260	0.681	-1.101	1.620	0.381	0.704	0.001	0.558
**Intercept Dog 22**	-1.338	1.148	-3.697	1.022	-1.165	0.254	-1.934	-1.037
**Angle of stimuli rotation Dog 22 (1)**	-0.142	1.161	-2.529	2.245	-0.122	0.904	-0.471	0.296
**Intercept Dog 29**	-2.266	0.971	-4.203	-0.329	-2.333	0.023	-2.605	-2.014
**Angle of stimuli rotation Dog 29 (1)**	2.410	0.968	0.478	4.341	2.488	0.015	2.085	2.755
**Intercept Dog 37**	-0.087	0.461	-1.006	0.832	-0.189	0.851	-0.211	0.070
**Angle of stimuli rotation Dog 37 (1)**	0.780	0.459	-0.135	1.696	1.701	0.094	0.614	0.953

(1) Z-transformed to a mean of 0 and a standard deviation of 1.
The columns Min and Max show the range of the estimates derived when excluding cases one at a time (DFBetas).

Table S11. All recruited dogs. Related to “STAR Methods/Subjects”.

dog ID	sex	age	breed	Previous TS experience	S+	pre-training sessions	training sessions
**1**	M	8	Border Collie	Yes	2A	6	3
**2**	M	10	Border Collie	Yes	1A	7	2
**3**	M	3	Mix	Yes	1B	2	0
**4**	M	8	Mix	Yes	1B	2	0
**5**	M	5	Border Collie	No	3B	1	0
**6**	M	4	Border Collie	Yes	1A	2	0
**7**	M	9	Border Collie (no pedigree)	Yes	2B	6	51
**8**	M	9	Poodle	Yes	3B	6	2
**9**	M	4	Australian Shepherd	Yes	3B	8	2
**10**	M	5	Border Collie	No	3A	12	127
**11**	F	9	Border Collie	Yes	3B	7	15
**12**	M	3	Border Collie	Yes	1A	8	45
**13**	F	10	Mix-Jack Russel Terrier	Yes	1B	8	45
**14**	M	12	Mix	Yes	1B	6	31
**15**	M	3	Canarian Warren Hound	Yes	2A	7	28
**16**	M	8	Mix	Yes	2B	7	14
**17**	F	6	Border Collie	Yes	3A	11	45
**18**	F	14	Mix-Chihuahua	Yes	3B	5	15
**19**	F	6	Mix-small Terrier	Yes	3B	7	11
**20**	M	8	Border Collie	Yes	2A	6	29
**21**	F	8	Border Collie	Yes	2B	7	57
**22**	M	3	Mix	No	2A	18	101
**23**	F	4	Australian Shepherd	Yes	2B	11	23
**24**	F	9	Border Collie	Yes	1A	5	45
**25**	F	7	Mix-small Terrier	Yes	3A	10	17
**26**	M	0.42	Border Collie	No	3A	7	40
**27**	F	11	Border Collie	Yes	3A	6	54
**28**	F	6	Australian Shepherd	No	1B	41	19
**29**	F	9	Mix	No	3A	29	70
**30**	F	10	Australian Shepherd	No	1B	21	30
**31**	F	11	Mix-Beagle/Terrier	Yes	2A	6	55
**32**	F	8	Mix-Whippet/Pinscher	Yes	1A	7	39
**33**	M	10	Berger des Pyrénées Á Face Rase	Yes	1A	6	45
**34**	M	2	Berger des Pyrénées Á Face Rase	Yes	1B	4	46
**35**	M	10	Border Collie	Yes	1B	5	45
**36**	M	4	Mix-small Terrier	No	3A	21	34
**37**	F	5	Border Collie	Yes	1A	11	40
**38**	F	9	Border Collie	Yes	1B	8	70

The age (at the beginning of the experiment) is in years. The column “Previous TS experience” shows whether dogs had already participated to a touchscreen experiment and therefore had been trained on the use of a touchscreen before the current experiment.
S+ stands for reinforced stimulus. The labels of the stimuli refer to Figure 5.
Pre-training sessions comprise both phases 1 and 2 described in the supplementary text. Training sessions comprise phases 3 and 4.

Table S12. Demographics of the 8 tested dogs. Related to “STAR Methods/Subjects”.

Dog ID	Sex	Breed	Age (years)	Previous touch screen experience	Sessions to test	Reinforced stimulus
**11**	F	Border Collie	9	Yes	15	3B
**21**	F	Border Collie	8	Yes	57	2B
**29**	F	Mongrel	9	No	70	3A
**37**	F	Border Collie	5	Yes	40	1A
**7**	M	Border Collie (no pedigree)	9	Yes	51	2B
**10**	M	Border Collie	5	No	127	3A
**20**	M	Border Collie	8	Yes	29	2A
**22**	M	Mongrel	3	No	101	2A

The column “Previous touch screen experience” shows whether each subject had already participated to a touchscreen experiment before the current one and therefore whether the dog was already trained for this purpose. The last column (“Sessions to test”) reports the number of training sessions each dog needed to meet the learning criteria and be tested. The labels of reinforced stimuli (last column) refer to Figure 5.
